# Evaluation of Oxidative Stress and Antioxidant Effects of Methylxanthines in Adult Zebrafish Exposed to Zinc Oxide Nanoparticles (ZnO-NPs)

**DOI:** 10.3390/medicina62010021

**Published:** 2025-12-22

**Authors:** Cristian Dan Pavel, Carmen Lidia Chiţescu, Oana-Maria Dragostin, Lorena Dediu, Iuliana Aprodu, Ancuţa Dinu Iacob, Rodica Vatcu, Alexandra-Simona Zamfir, Carmen Lăcrămioara Zamfir

**Affiliations:** 1 Department of Morpho-Functional Sciences I, “Grigore T. Popa” University of Medicine and Pharmacy, 700115 Iaşi, Romania; cristian-dan_g_pavel@d.umfiasi.ro (C.D.P.); carmen.zamfir@umfiasi.ro (C.L.Z.); 2 Department of Pharmaceutical Sciences, Research Centre in the Medical-Pharmaceutical Field, Faculty of Medicine and Pharmacy, “Dunărea de Jos” University of Galati, 800008 Galaţi, Romania; oana.dragostin@ugal.ro (O.-M.D.); ancuta.dinu@ugal.ro (A.D.I.); rodica.vatcu@gmail.com (R.V.); 3 Faculty of Food Science and Engineering, ‘’Dunărea de Jos’’ University of Galati, 111 Domnească Street, 800201 Galaţi, Romania; lorena.dediu@ugal.ro (L.D.); iuliana.aprodu@ugal.ro (I.A.); 4 Department of Medical Sciences III, “Grigore T. Popa” University of Medicine and Pharmacy, 16 Universității Street, 700115 Iași, Romania; simona-zamfir@umfiasi.ro

**Keywords:** antioxidant defense system, molecular docking, redox homeostasis, protective potential

## Abstract

*Background and Objectives:* Oxidative stress plays a central role in numerous pathological and toxicological processes, and in vivo investigations are essential for understanding integrated systemic responses. Methylxanthines have been reported to modulate redox homeostasis through multiple mechanisms, but their effects in aquatic vertebrate models under metal nanoparticle-induced oxidative stress remain poorly characterized. *Materials and Methods:* In the present study, adult zebrafish were exposed for 15 days to ZnO nanoparticles (0.69 mg/L) as a pro-oxidant model, and to methylxanthines (caffeine, theobromine, theophylline; 50 mg/L). Oxidative stress biomarkers were assessed by measuring the levels of glutathione peroxidase 1 (GPx1), catalase (CAT), superoxide dismutase (SOD), and reduced glutathione (GSH) in whole-body homogenates using ELISA. Complementary molecular docking was performed to investigate methylxanthine–enzyme interactions. *Results:* The most substantial change was observed for SOD level, which significant increased compared to the control group (from 0.122 to 1.090 ng/g; *p* = 0.001), followed by CAT, which rose from 38.3 pg/g to 100.8 pg/g; *p* = 0.001), and GPX1 which increased from 84.3 pg/g to 142.2 pg/g; *p* = 0.011). In parallel, GSH levels decreased by 58.7% (*p* = 0.001). Co-exposure to methylxanthines significantly modulated the ZnO-NPs exposure response, by mitigating the increase in antioxidant enzyme levels and restoring glutathione. Among the tested compounds, theobromine exerted the strongest protective effect on GPx1 and GSH and caffeine primarily influenced CAT and SOD, whereas theophylline showed overall weaker responses. The molecular docking investigation indicated that all tested methylxanthines can attach to different cavities of the antioxidant enzymes. Theophylline and theobromine established hydrogen bonds and π-stacking interactions with the interfacing amino acids, potentially contributing to the modulation of enzymes stabilization and function under physiological conditions. *Conclusions:* ZnO-NPs trigger a robust systemic response in zebrafish, whereas methylxanthines display distinct compound-specific modulating effects.

## 1. Introduction

Oxidative stress refers to an imbalance between the production and elimination of reactive oxygen species (ROS), highly reactive oxygen-derived molecules generated mainly during mitochondrial respiration and other cellular processes [1,2,3].

Under physiological conditions, ROS play essential roles in cellular signaling and regulate metabolic and immune functions, including activation of various transcription factors, cellular differentiation, and apoptosis [4,5]. However, their excessive accumulation—triggered by environmental exposures, lifestyle factors, pathological conditions, chronic inflammation, or metabolic disorders [6,7,8,9]—overwhelms antioxidant defenses and leads to biomolecular damage, affecting protein integrity [2,3,10], promoting lipid peroxidation [9,10], and inducing DNA lesions associated with genomic instability, aging, and carcinogenesis [6,11,12].

ROS have been increasingly studied in biology and medicine due to their central role in the pathogenesis of numerous diseases. These include gastrointestinal [13] and hepatic disorders [14], renal dysfunction [15], infertility [16], diabetes [10], neurodegenerative conditions [1], cancer [17,18], cardiovascular disease [10,19], atherosclerosis [10,20], inflammatory bowel disease [13,18], and metabolic syndrome [10,21].

To mitigate oxidative damage, cells rely on an intricate antioxidant defense system involving both enzymatic and non-enzymatic components [22]. Superoxide dismutase (SOD), catalase (CAT), and glutathione peroxidase (GPx) act sequentially as enzymatic antioxidants to neutralize ROS [23,24,25]. Non-enzymatic antioxidants including reduced glutathione (GSH), thioredoxin (Trx), melatonin, and irisin complement enzymatic defenses by directly scavenging free radicals and supporting redox cycling [26,27].

A variety of experimental models have been developed to investigate ROS-mediated damage, allowing for the controlled assessment of molecular, cellular, and tissue-level effects of oxidative imbalance [11,28,29,30]. Among them, rodent models [30,31], zebrafish models using embryos or adult fish [11,28,32], and cell lines [33,34] are commonly employed, enabling the evaluation of redox-sensitive pathways and antioxidant defense mechanisms.

Several studies support the use of the zebrafish (*Danio rerio)* model for investigating oxidative stress, demonstrating its capacity to exhibit measurable and reproducible responses to pro-oxidant agents. Kim et al. (2021) employed hydrogen peroxide (H_2_O_2_) to trigger oxidative stress and confirmed the model’s responsiveness to oxidative injury [34]. Cheng et al. (2025) further confirmed this by showing that zebrafish embryos exposed to environmental toxicants such as chlorinated and methylated p-benzoquinones exhibited changes in antioxidant gene expression [35]. Other compounds, including bisphenol [29], paclobutrazol [36], chloramine [37], and aluminum [38], have also been employed to induce oxidative stress in zebrafish, highlighting the relevance of this model for translational research.

Metal-based nanoparticles, including ZnO-NPs, induce oxidative stress through multiple complementary mechanisms. One mechanism involves the release of metal ions at the nanoparticle surface, which engage in Fenton-like reactions and generate hydroxyl radicals, initiating ROS formation [39]. A second pathway entails direct interactions of ZnO-NPs with cellular structures such as mitochondria and plasma membrane NADPH oxidases, disrupting the electron transport chain and further enhancing ROS accumulation [11].

The compensatory response, represented by the activation and upregulation of antioxidant enzymes, is progressively followed by the suppression of antioxidant defenses, the induction of apoptosis, and alterations in the expression of antioxidant enzyme-related genes [36,39,40]. A third mechanism involves an inflammatory cascade initiated by ROS overproduction, which stimulates pro-inflammatory signaling pathways (TNF-α, IL-6, NF-κB) [39] and promotes additional ROS generation from immune cells, thereby amplifying oxidative pressure and contributing to genotoxic damage such as DNA fragmentation and lipid peroxidation [28,41].

In addition, partial intracellular solubilization of ZnO-NPs results in Zn^2+^ release, which interferes with the transcriptional regulation and functionality of antioxidant enzymes, explaining the frequently observed biochemical pattern of early enzyme activation followed by subsequent inhibition [42,43]. As oxidative pressure persists, an initial compensatory antioxidant response is followed by the suppression of antioxidant defenses, apoptosis induction, and alterations in the expression of antioxidant enzyme-related genes.

Numerous natural compounds have been examined for in vitro and in vivo antioxidant capacity [1,20,28]. Methylxanthines have also been shown to exert antioxidant effects through several mechanisms, including modulation of redox-sensitive signaling pathways (e.g., Nrf2/ARE), inhibition of lipid peroxidation, free radical scavenging, and anti-inflammatory activity [29,30,31,32]. There is evidence supporting the ability of methylxanthines to protect against oxidative stress in cellular [44,45], animal [46], and human models [47].

The present study employs the vertebrate model *Danio rerio* (zebrafish) to evaluate biochemical alterations associated with experimental exposure to zinc oxide nanoparticles (ZnO-NPs). The study focuses on GSH, CAT, SOD, and GPx1, biomarkers widely used to monitor early redox-related responses and antioxidant modulation in nanoparticle-exposed organisms [30,48].

Although methylxanthines are recognized modulators of oxidative signaling [49,50], studies on in vivo models, particularly in the context of ZnO-NPs exposure, remain limited. Given their distinct methylation patterns and differential biological activities, caffeine, theobromine, and theophylline may exert compound-specific effects on redox homeostasis, supporting the rationale for their comparative evaluation in this context.

A complementary molecular docking analysis aimed at exploring the potential interactions of the methylxanthines with the antioxidant enzymes was also performed, offering mechanistic insights that may help explain the differential biochemical responses observed in vivo. Histopathological aspects will be addressed in a separate, follow-up manuscript.

## 2. Materials and Methods

### 2.1. Synthesis of Zinc Oxide Nanoparticles (ZnO-NPs)

Zinc acetate was dissolved in ethanol at a concentration of 3.35 mM by stirring for 30 min at 60 °C and 200 RPM. In parallel, a potassium hydroxide solution (0.4 g/L) was prepared. The potassium hydroxide solution was added dropwise to the zinc acetate solution under continuous stirring for one hour. The resulting suspension was left to rest for 2 h, followed by centrifugation at 10,000 RPM for 10 min. The obtained ZnO-NPs were washed with ethanol, dried in an oven, and stored in tightly sealed amber vials until further use. ZnO-NPs were synthesized in our laboratory following a validated protocol widely applied in related research [43,51,52,53].

### 2.2. Experimental Design of the In Vivo Experiment

Adult zebrafish (*Danio rerio*) of both sexes, aged 6 months, were acclimated for two weeks after purchase and prior to the start of the experiment. Based on experimental designs reported in the literature for similar toxicological studies on adult zebrafish [28,37,54], the fish were further randomly allocated into eight experimental groups (*n* = 15 per group), each organized in triplicate (five fish per replicate, housed in separate tanks). Fishes were fed daily with commercial feed and maintained in aquaria at 28 ± 1 °C under a 14 h light/10 h dark photoperiod. The pH value, hardness, temperature, and dissolved oxygen of tap water were 7.34–7.62, 95–112 mg/L as CaCO_3_, 24 ± 1 °C, and above 7.2 mg/L, respectively.

As the available literature on ZnO-NP exposure in zebrafish is highly heterogeneous, with substantial variability in experimental doses, exposure durations, and biological endpoints, no general consensus exists regarding a standard exposure level. This lack of standardization is reflected in published studies, where ZnO-NP concentrations vary from sublethal doses used to investigate metabolic and antioxidant responses [11,55,56] to higher concentrations designed to assess acute toxicity or tissue-specific damage [43,57,58]. In this context, the dose selected in the present study falls within the sublethal range previously reported for adult zebrafish models and was therefore considered adequate to elicit measurable biological responses without inducing overt toxicity [11,55,56].

Group 1 received no treatment (untreated control), while groups 2–5 were exposed daily for 15 days to 1/5 of the estimated LC_50_ for ZnO-NPs (0.69 mg/L in water) according to Al-Zahaby S.A. et al. (2023) [11].

Groups 3–5 were similarly exposed to ZnO-NPs for 15 days, while simultaneously receiving daily administration of one of the three methylxanthines (caffeine, theophylline, or theobromine, each at 50 mg/L), in order to assess their potential protective effects. Groups 6–8 received only methylxanthines (50 mg/L for 15 days) under the same experimental conditions, without prior ZnO-NPs exposure (Table 1).

Throughout the experimental period, water in each tank was renewed daily, and the appropriate concentrations of ZnO-NPs or xanthine-derived compounds were added from stock solutions in the replacement water.

The concentration of 50 mg/L for methylxanthines was selected in accordance with the existing literature on zebrafish, and has been shown to produce detectable physiological outcomes in adult zebrafish, while remaining below toxic thresholds [59,60]. This dose enables the observation of measurable biological responses while ensuring the safety of the experimental model and comparability with other pharmacological studies [61,62]. As for theobromine and theophylline, the available literature is limited; in the present study, similar concentrations were employed to obtain pharmacological effects without mortality.

At the end of the experiment, the fish were euthanized by gradual cooling in crushed-ice water, a method considered conditionally acceptable for small fish species according to the AVMA Guidelines for the Euthanasia of Animals, (2020) [63].

The study was approved by the Ethics Committee of “Gr. T. Popa”, Medicine and Pharmacy University, Iaşi, Romania, and all methods were carried out in accordance with relevant guidelines and regulations (Approval number: 325/15 June 2023). This study was carried out in compliance with the ARRIVE (Animal Research: Reporting of In Vivo Experiments) guidelines [64].

### 2.3. Sample Preparation and Bicochemical Analysis

Biochemical determinations were performed on tissue extracts. Whole-body homogenate supernatant from zebrafish was obtained by homogenization, followed by centrifugation (10,000 g, 15 min). The supernatant was stored at −20 °C until analysis. The levels of glutathione peroxidase 1 (GPx1), catalase (CAT), superoxide dismutase (SOD), and reduced glutathione (GSH) were quantified using standardized research ELISA kits (ELK Biotechnology, Sugar Land, TX, USA), specifically validated for *Danio rerio* (zebrafish), according to the manufacturers’ instructions. The assays are based on competitive (for GSH, catalog no ELK10846 ELK Biotechnology, USA) or sandwich (for: GPx1, catalog no ELK8862; SOD, catalog no ELK8859; CAT, catalog no ELK8860) immunoenzymatic methods. All ELISA measurements were performed using a semi-automated microplate analyzer (UT6500, MRC, Holon, Israel). Tissue homogenates were analyzed at a fixed dilution factor of 1:5. Detailed procedures are provided in the Supplementary Materials. Normalizing enzymatic biomarkers to wet body mass has been considered appropriate in evaluating systemic physiological responses [65]. Therefore, in the present study, the concentrations obtained by analysis were normalized to fish body mass (wet weight), and results were expressed as amount per g wet tissue.

### 2.4. Statistical Analysis

The Shapiro–Wilk test was applied to assess the normality of data distribution, while Levene’s test was used to verify the homogeneity of variances across groups. In cases of variance heterogeneity (*p* ≤ 0.05), data transformations were applied to stabilize variability. Statistical significance was evaluated by one-way ANOVA followed by Tukey’s and Dunnett’s post hoc tests, or by non-parametric Kruskal–Wallis analysis with Dunn’s multiple comparisons for non-normally distributed data. Differences were considered statistically significant at *p* < 0.05. Results are expressed as mean ± standard deviation (SD) or as median and interquartile range (IQR). Correlations between variables, as well as their interrelationships, were examined using correlation tests, regression analysis, and principal component analysis (PCA). Hierarchical cluster analysis (HCA) was performed to identify patterns of similarity among experimental groups. Statistical analyses were performed with XLSTAT software, version 2023.3.0.

### 2.5. Molecular Docking Tests

The AutoDock Vina 1.2.0 on the SwissDock [66,67] was used to perform molecular docking tests, such as to identify atomic level interaction particularities between each methylxanthine and the antioxidant enzymes considered in the experimental approach. The models of GPx1 (2F8A), [68], CAT (7P8W) [69], and SOD (2JLP) [70] enzymes, used as receptors in the molecular docking procedure, were selected from the RCSB Protein Data Bank [71]. The PDBeChem database was interrogated for retrieving the models of the caffeine, theobromine, and theophylline ligands.

The top-scoring three-dimensional model of each type of antioxidant enzyme-methylxanthine complex, as recommended by AutoDock Vina ranking based on the interaction energy values, was further characterized using PDBePISA [72] and VMD 1.9.3. software [73].

## 3. Results

### 3.1. Comparative Analyses of Antioxidant Response Markers

To provide an overview of the antioxidant profile, the immunoreactive concentrations of GPx1, catalase, SOD, and GSH measured in the whole-body homogenate supernatants are summarized in Table 2. Data are expressed as median (interquartile range, IQR) for variables not normally distributed (GPx1, SOD, GSH) and as mean ± standard deviation (SD) for normally distributed variables (CAT).

**Figure 1:**
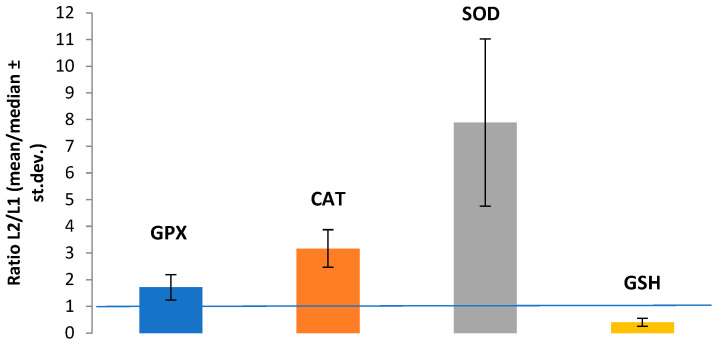
Relative changes compared with the control group (L1) expressed as the L2/L1 ratio. Data are expressed as medians for concentrations of GPX1, SOD, and GSH (non-normal distributions) and as mean for CAT (normal distribution confirmed).

Compared with the control group (L1), exposure to ZnO-NPs (L2) induced marked changes in all biomarkers (Figure 1). The analysis of L2/L1 ratios shows that SOD, GPX1, and CAT exhibited increased values relative to baseline (ratio >1), indicating activation of antioxidant mechanisms. SOD displayed a marked increase, with high variability across samples (large standard deviation), due to a heterogeneous response. In contrast, GSH showed a ratio <1, reflecting decreased levels.

Normality and variance homogeneity were confirmed only for CAT (Shapiro–Wilk *p* > 0.05; Levene *p* = 0.542), while SOD (Shapiro–Wilk *p* < 0.05; Levene *p* = 0.001), GPX1 (Shapiro–Wilk *p* < 0.05; Levene *p* = 0.006) and GSH (Shapiro–Wilk *p* < 0.05; Levene *p* < 0.001) did not meet these assumptions, thus requiring non-parametric analysis for subsequent group comparisons.

To evaluate the antioxidant response in groups 2–8 compared to the control group (L1), univariate tests (ANOVA or Kruskal–Wallis with post hoc multiple comparisons when the assumptions of normality and homogeneity of variances were not met) were applied to GPX1, CAT, SOD, and GSH levels. Results for each marker are presented separately.

**Figure 2:**
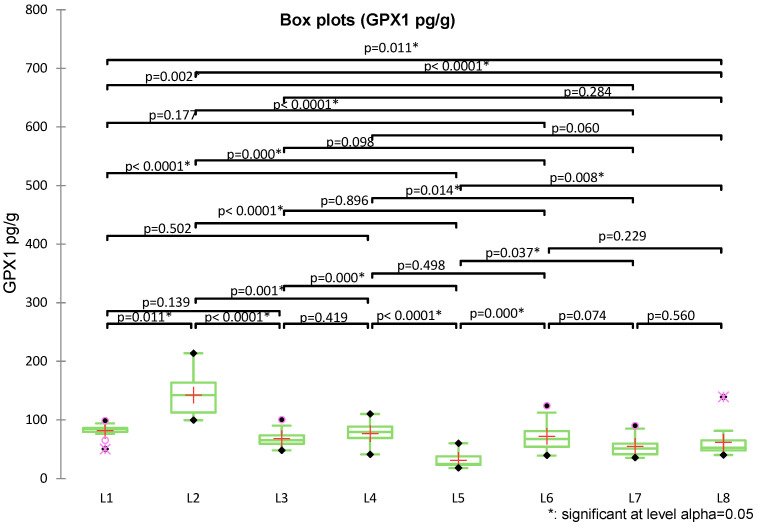
Box-plot of the Kruskal–Wallis analysis with Dunn/Holm post hoc multiple comparisons of GPX1 concentrations between groups L1–L8.

Regarding GPX1 levels, Kruskal–Wallis analysis followed by Dunn–Holm post hoc tests revealed significant differences among groups (pg/mL) (Figure 2). Exposure to ZnO-NPs in the untreated group (L2) induced a significant increase in GPX1 compared with the control (L1, *p* = 0.011). Co-exposure with methylxanthines (L3–L5) significantly reduced GPX1 levels compared with ZnO-NPs alone (*p* < 0.001–0.001), due to a possible protective effect of caffeine, theophylline, and theobromine. Groups exposed exclusively to methylxanthines (L6–L8) did not differ significantly from control (*p* > 0.05), but were clearly distinct from the ZnO-NPs group (*p* = 0.000–0.037).

**Figure 3:**
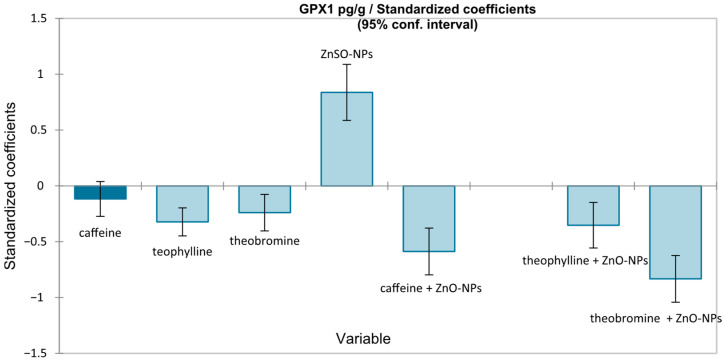
Graphical representation of standardized coefficients obtained from multiple linear regression, showing the impact of independent variables on GPX1 (pg/g wet tissue).

A multiple linear regression was then performed with GPX1 as the dependent variable and methylxanthines (caffeine, theophylline, theobromine), ZnO-NPs exposure, and their interactions as explanatory variables, using heteroscedasticity-corrected estimates. The overall model was significant (F(7112) = 33.956, *p* < 0.0001), indicating that the included predictors accounted for a substantial proportion of GPX1 variance (Figure 3). Regression analysis confirmed the role of ZnO-NPs exposure in increasing GPX1 (β = +0.838, *p* < 0.0001). Caffeine and theobromine significantly interacted with ZnO-NP exposure to reduce GPX1 levels, with a moderate protective effect for caffeine (β = −0.587, *p* < 0.0001) and the strongest effect for theobromine (β = −0.833, *p* < 0.0001).

**Figure 4:**
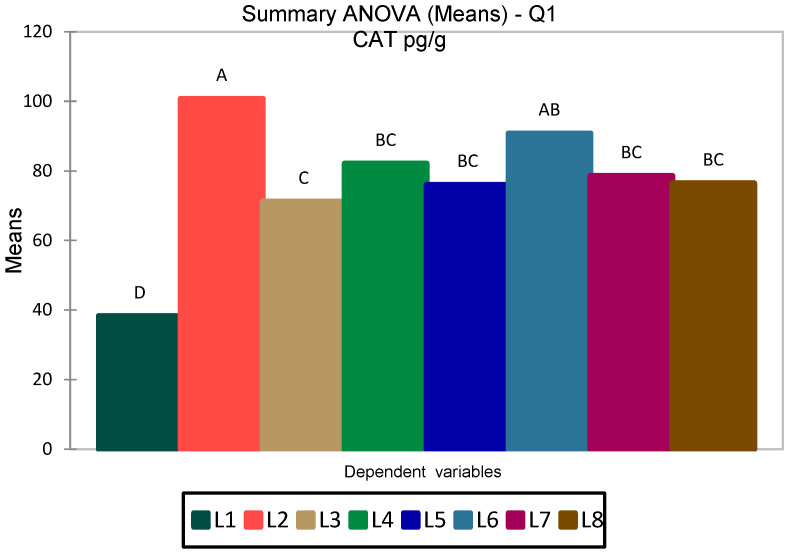
ANOVA analysis of CAT levels (pg/mL) in the eight experimental groups; values are expressed as means with 95% confidence intervals. Different letters above the bars indicate statistically significant differences between groups according to post hoc tests (Tukey HSD/Duncan, *p* < 0.05).

Between-group comparisons for CAT were performed using one-way ANOVA, as the data followed a normal distribution. The overall model was significant: F(7112) = 14.561, *p* < 0.0001, with R^2^ = 0.476 (adjusted R^2^ = 0.444) (Figure 4).

Post hoc analyses confirmed significant differences between groups (Table S1), which are also graphically represented in Figure 4. The Tukey HSD test showed significantly higher CAT levels in the L2 group (ZnO-NPs, 100.7 pg/g) compared with all other groups, with a particularly marked difference relative to the control (L1, 38.3 pg/g; *p* < 0.0001). Additional differences were detected between L2 and L3–L5, as well as between L7 and L1. Duncan’s test confirmed this pattern, grouping the experimental sets into four distinct categories: L2 in class A (highest values), L6 and partly L4–L7 in intermediate classes (B–C), and the control (L1) in class D (lowest values) (Figure 4). Moreover, Dunnett’s test, using L1 as a reference, indicated significantly higher CAT levels across all other groups (L2–L8, *p* < 0.0001).

**Figure 5:**
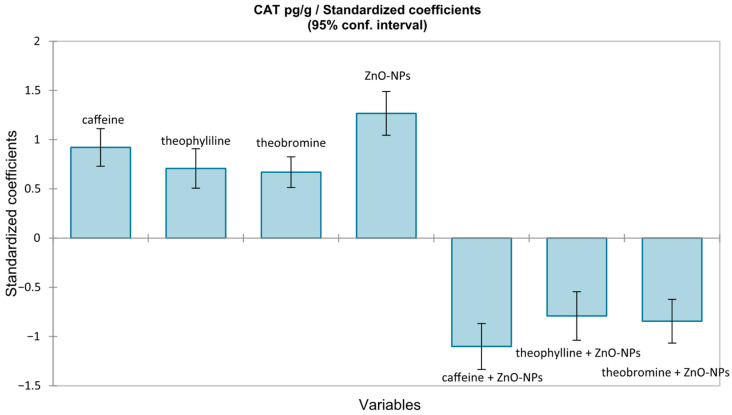
Standardized coefficients (β) of the multiple regression model for CAT (pg/g), with 95% confidence intervals.

A multiple linear regression model, using CAT as the dependent variable, was performed. Predictors were included as indicator variables (0/1) for caffeine, theophylline, theobromine, and ZnO-NPs exposure, together with their interaction terms; the intercept corresponded to the control group (L1), so that coefficients represent mean differences from the control, adjusted for the other factors. The overall model was significant (F(7112) = 14.561, *p* < 0.0001), (Figure 5). Exposure to ZnO-NPs was the strongest positive determinant of catalase levels (β = +1.27). In contrast, interactions of xanthine with ZnO-NPs exposure exerted inhibitory effects, strongest for caffeine (β = −1.10), followed by theobromine (β = −0.84) and theophylline (β = −0.79). However, methylxanthines themselves induced a moderate increase in catalase (groups 6–8), indicating an intrinsic activation of antioxidant mechanisms.

**Figure 6:**
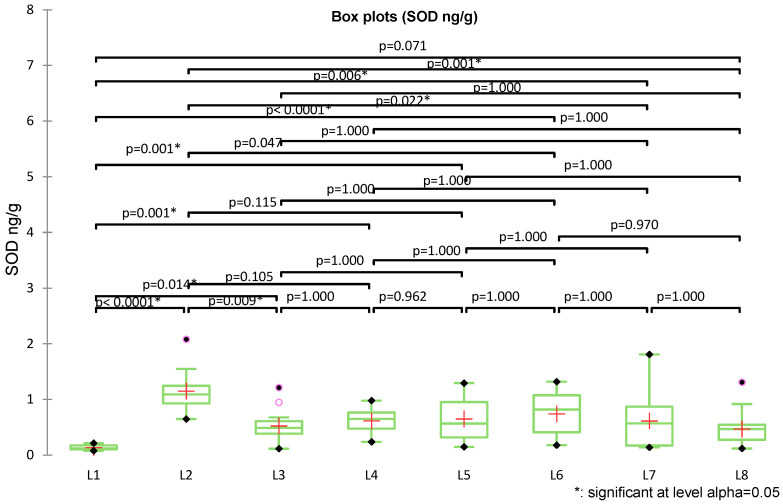
Box-plot representation of SOD levels (ng/g) across the eight experimental groups, with post hoc Dunn comparisons according to the non-parametric Kruskal–Wallis test.

SOD levels (ng/g) were determined in all eight experimental groups (n = 120). The overall mean was 0.613 ± 0.418 ng/g, ranging from 0.080 to 2.08 ng/g. Descriptive analysis by groups revealed substantial variability, with the highest levels observed in L2 (ZnO-NPs alone) and the lowest in the control group (L1). The Kruskal–Wallis test revealed significant differences among the experimental groups (*K* = 52.755, *df* = 7, *p* < 0.0001), confirming that SOD distributions differed significantly between groups (Figure 6).

The control group (L1) showed significantly lower SOD levels compared with all other groups, with highly significant differences (e.g., L1 vs. L2, *p* < 0.0001; L1 vs. L3, *p* = 0.014; L1 vs. L4, *p* = 0.001). L2 (ZnO-NPs) displayed the highest levels, differing significantly from L1 (*p* < 0.0001) as well as from L3 (*p* = 0.009) and L4 (*p* = 0.006), but also from L6 (*p* = 0.047), L7 (*p* = 0.022), and L8 (*p* = 0.001). Intermediate groups (L3–L5) had higher values than the control group L1 but did not differ significantly among themselves, whereas L6–L7 exhibited significantly different values compared to L1 (*p* < 0.001 and *p* = 0.006, respectively). The group treated with theobromine (L8) did not differ significantly from the control (*p* = 0.071).

**Figure 7:**
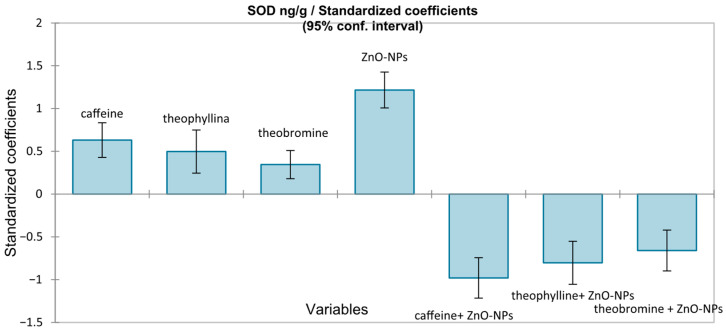
Standardized coefficients (β) from the multiple linear regression for SOD (ng/g), with 95% confidence intervals.

The multiple linear regression model predicting SOD was globally significant (F(7112) = 10.91, *p* < 0.0001), explaining about 37% of the variability (adjusted R^2^ = 0.368). Standardized coefficients showed ZnO-NPs exposure as the main positive determinant of SOD induction (β = +1.217, *p* < 0.0001), followed by moderate stimulatory effects of caffeine (β = +0.631), theophylline (β = +0.498), and theobromine (β = +0.345) (*p* < 0.0001). In contrast, methylxanthines had strong inhibitory effects: theophylline (β = −1.031), caffeine (β = −0.979), and theobromine (β = −0.659) (all *p* < 0.0001) showed a suppression of the stress-induced enzymatic response, with protective effects ranked as theophylline > caffeine > theobromine (Figure 7).

**Figure 8:**
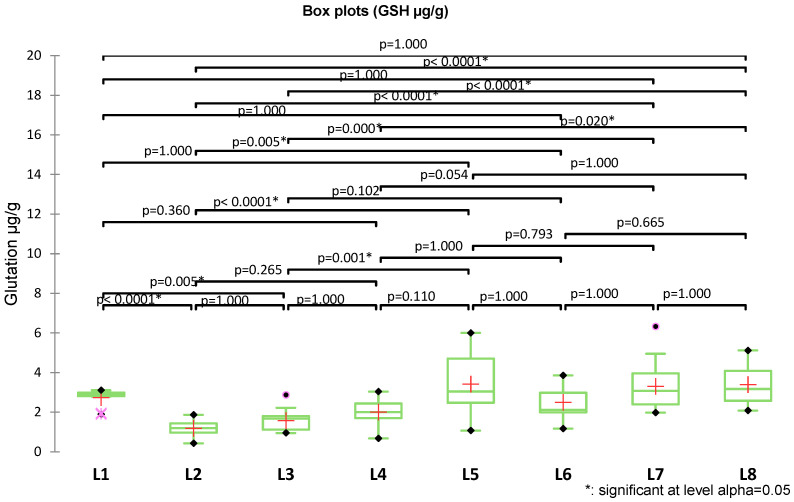
Box-plot representation of GSH levels across the eight experimental groups (L1–L8), analyzed using the Kruskal–Wallis test with Dunn post hoc comparisons.

Another parameter investigated was glutathione (GSH), as a central component molecule in cellular defense against oxidative stress. Variability was higher in groups with elevated levels (higher SD in L6–L8). Box-plots showed a progressive increase in GSH in exposed groups compared with the control, with maximum values in L7 and L8 (up to 6.3 µg/g), due to the influence of the experimental exposures (Figure 8).

Pairwise comparisons were performed using Dunn’s test with multiple comparison correction (Figure 8). The L2 group exhibited the lowest GSH levels, with values significantly reduced compared to almost all other groups (e.g., L1, L5, L7, and L8; all *p* < 0.0001). GSH levels in L3 were moderately reduced and significantly lower than those in L1 (*p* = 0.005), L5 (*p* = 0.001), and particularly L7 and L8 (both *p* < 0.0001). The highest levels were observed in L7 and L8, which differed significantly from the groups, L2–L4. Overall, the analysis demonstrates a gradual increase in glutathione, with the L2 at the minimum, L4–L6 at intermediate levels, and L7–L8 at the maximum.

**Figure 9:**
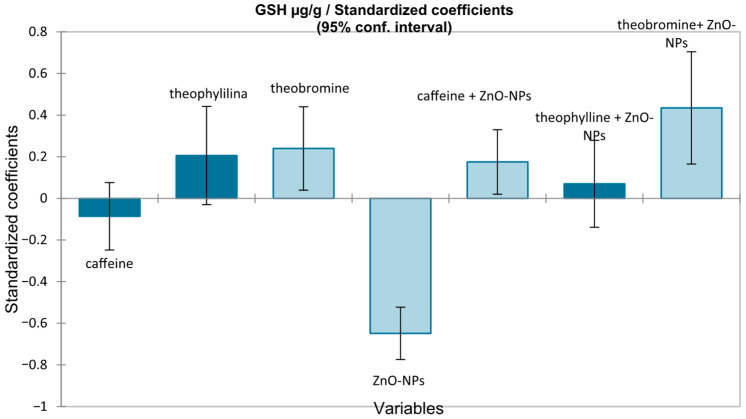
Standardized coefficients (β) from multiple linear regressions with robust HC0 errors for the prediction of glutathione levels (µg/mL), with 95% confidence intervals.

For the analysis of GSH, a multiple linear regression model was applied, including caffeine, theophylline, theobromine, ZnO-NPs exposure, and their interactions as predictors (Figure 9).

As Levene’s test indicated heterogeneity of variances (F = 4.906, *p* < 0.0001), robust HC0 (White) standard errors were used. The overall model was significant (F(7112) = 13.192, *p* < 0.0001), explaining ~42% of GSH variability (adjusted R^2^ = 0.418). ZnO-NPs exposure was the strongest negative determinant (β = −1.55 µg/g; standardized β = −0.648, *p* < 0.001). Among methylxanthines, theobromine showed the most consistent protective effect, both directly (β = +0.66 µg/g, *p* = 0.019) and in interaction with ZnO-NPs exposure (β = +1.56 µg/g; standardized β = +0.44, *p* = 0.002). Caffeine exerted a weaker but detectable protective effect only under ZnO-NPs exposure (β = +0.63 µg/g, *p* = 0.027), whereas theophylline was not significant.

### 3.2. Correlation Between Analyzed Parameters

To examine potential associations among the measured redox-related biomarkers, Spearman correlation analysis was performed for GPX1, GSH, CAT, and SOD (Table S2 and Figure S1). A negative correlation was observed between glutathione and GPX1 (r^ss^; = −0.482, *p* < 0.0001), reflecting compensatory mechanisms between these markers. Glutathione also showed negative correlations with SOD (r^ss^; = −0.374, *p* < 0.0001) and CAT (r^ss^; = −0.190, *p* = 0.038), indicating coordinated adjustments between non-enzymatic and enzymatic antioxidant components under ZnO-NPs exposure. CAT and SOD were strongly and positively correlated (r^ss^; = 0.399, *p* < 0.0001), consistent with their functional interdependence as complementary enzymatic defenses.

To further explore the relationships between biomarkers, multiple linear regression analyses were performed, considering each marker in turn as the dependent variable and the others as predictors.

All models were significant (*p* < 0.0001), with explained variance ranging from 16% to 28%. GPX1 variability was driven mainly by GSH, which exerted a strong negative effect (β = −0.466, *p* < 0.0001), while CAT and SOD had no meaningful contribution (Figure S2). Conversely, GSH was negatively predicted by GPX1 (βstd = −0.467, *p* < 0.0001) and SOD (βstd = −0.177, *p* = 0.030), but not by CAT. For CAT, SOD emerged as the dominant positive predictor (βstd = 0.414, *p* < 0.0001), consistent with their functional interdependence, whereas GPX1 and GSH were not significant. Finally, SOD was best explained by CAT (βstd = 0.383, *p* < 0.0001), confirming their complementarity, while GSH contributed negatively (βstd = −0.185, *p* = 0.050) and GPX1 only marginally.

These findings highlight the functional interdependence between CAT and SOD, along with the compensatory inverse role of GSH relative to GPX1 and SOD, outlining an integrated antioxidant response.

**Figure 10:**
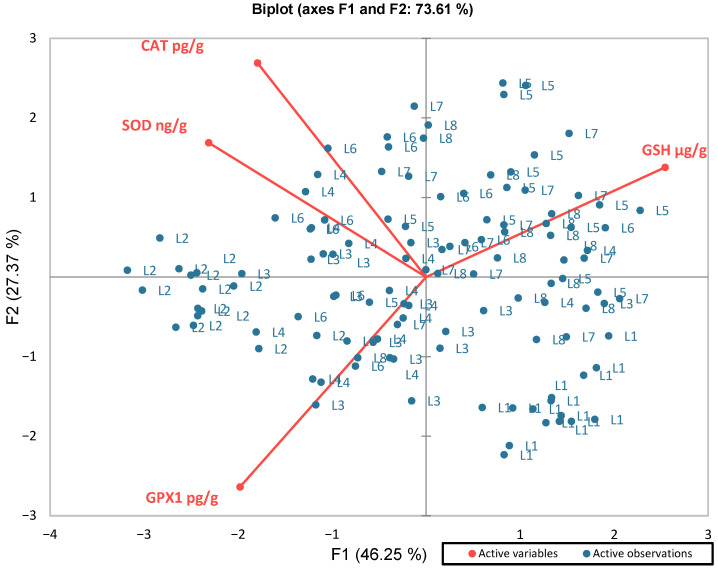
Principal component analysis (PCA) of antioxidant markers (GPX1, GHS, CAT, and SOD).

To complement these regression and correlation analyses, principal component analysis (PCA) was applied to provide an integrative view of the relationships among the markers (Figure 10). The first two principal components (F1 and F2) together explained 73.61% of the total variance (F1 = 46.25%, F2 = 27.37%), indicating a robust model for interpretation.

Variable loadings revealed two major axes of variability. F1 clearly opposed GPX1 (negative projection) to glutathione (positive projection), reflecting the inverse relationship already observed in regression models. In contrast, F2 was mainly driven by catalase and SOD, both showing positive and correlated projections, consistent with their functional cooperation in hydrogen peroxide removal. Thus, PCA distinguished two complementary dimensions of the defense: a GPX1–GSH axis, indicative of glutathione turnover under ZnO-NPs exposure, and a CAT–SOD axis, reflecting enzymatic coordination in ROS neutralization.

Hierarchical cluster analysis of all measured parameters (GPX1, CAT, SOD, and GSH) separated the experimental groups into five clusters (Figure S3). The ZnO-NPs group (L2) formed a distinct cluster, indicating a specific response pattern. Methylxanthines administered alone (L6–L8) was grouped together and in proximity to the control (L1), consistent with the absence marked changes in antioxidant biomarkers. The ZnO-NPs–methylxanthine co-exposure groups (L3–L5) displayed an intermediate clustering pattern, reflecting a modulation of the ZnO-NPs effect.

### 3.3. Molecular Docking Analysis

Molecular docking investigations were further carried out to check the ability of the methylxanthines to interact with the enzymes. The results presented in Table 3 indicated that all tested ligands are able to attach with different affinities to different sites of the enzymes.

**Figure 11:**
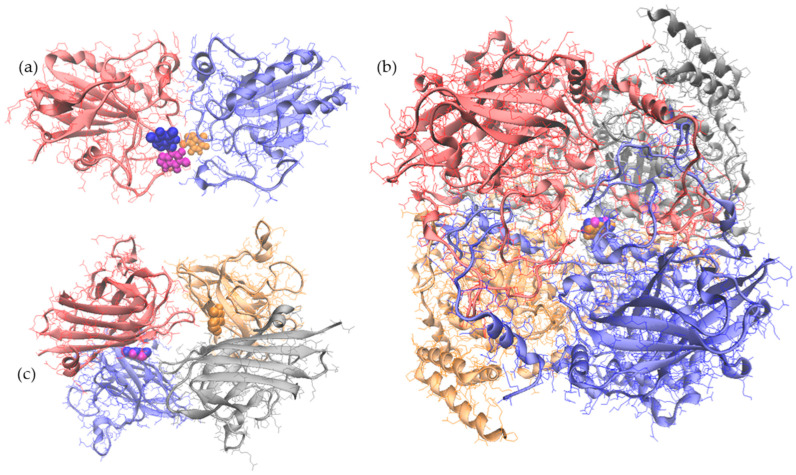
Three-dimensional models of the complexes resulting from molecular docking, in which glutathione peroxidase 1 (**a**), catalase (**b**), and superoxide dismutase (**c**) were used as receptors for caffeine (blue), theophylline (magenta), and theobromine (orange).

No important differences in terms of binding energy were noticed between complexes formed by GPx1 with the tested ligands (Table 3). The caffeine molecule preferentially attached to a small site located on chain B (Figure 11), the interaction surface between the two molecules of the complex being 78.6 Å^2^. Among the GPx1 amino acids in direct contact with the ligand, Gln^78^ is the only residue with the potential to be involved in establishing hydrophobic contacts. Unlike the caffeine molecule, theobromine and theophylline preferred different binding sites, directly interfacing amino acids of the two chains of the enzyme. Indeed, the GPx1 molecule involved a significantly larger surface of 132.2 and 124.4 Å^2^ in the interaction with theophylline and theobromine, respectively, (Table 3). Based on the solvation free energy values (ΔGi) measured when interfacing the theophylline molecule with each enzyme monomer, it appeared that the ligand has better affinity towards chain A (ΔGi of −0.1 kcal/mol) compared to chain B (ΔGi of 0.4 kcal/mol). Moreover, a hydrogen bond with the Thr^149^ from chain B is involved in stabilizing the GPx1- theophylline complex. Among the residues interfacing the ligand within the complex, the highest contribution to the hydrophobic effect was noticed in the case of Lys^146^ from chain A and Gln^78^ from chain B. Regarding the complex formed with theobromine, the free energy gain of −0.7 kcal/mol registered upon the assembly formation indicates the existence of favoring hydrophobic interfaces and positive enzyme–ligand affinity. The enzyme–ligand surface is additionally stabilized through interactions involving amino acids of the chain A, namely a hydrogen bond with Thr^149^ and one π-stacking interaction with His^81^ (A). Nevertheless, given the negative value of the GPx1-theobromine complex dissociation free energy (ΔG_diss_ of −1.1 kcal/mol), the complex appears rather unstable from the thermodynamic point of view. Lys^146^ and Leu^147^ residues have solvation energy, which indicates an important contribution to the hydrophobic effect, and therefore to the protein–ligand aggregation in an aqueous environment.

As a heme-containing tetrameric enzyme, CAT is involved in the defense against oxidative damage though the removal of hydrogen peroxide molecules, which are turned into water and oxygen through a two-step reaction mechanism involving the heme [74]. All tested ligands preferentially attached to the same binding site, located in the hydrophobic core defined at the confluence of the four monomers (Figure 11). As the result of structural differences among ligands and their relative orientation in respect to the binding site, the receptor–ligand interaction surface ranged between 171.5 and 174.1 Å^2^ (Table 2). The ligands directly interfaced amino acids of the four monomers of the enzyme, located near the heme active site. The strongest binding energy values were noted for the complexes formed with theobromine (−5.609 kcal/mol) and theophylline (−5.522 kcal/mol). In fact, the CAT interaction with theobromine involved two hydrogen bonds with His^364^ (chain C) and Arg^66^ (chain D), whereas the interface with theophylline was stabilized through one hydrogen bond with Arg^66^ (chain B). In any case, no hydrophobic interfaces or positive protein affinity were observed, as ΔGi was 0.5 kcal/mol in the case of CAT- theophylline complex, and 1.7 kcal/mol in the case of the complexes formed with theobromine and caffeine.

SOD is a heteromeric Cu-Zn enzyme, which ensures the dismutation of superoxide into hydrogen peroxide and oxygen by relying on a catalytic mechanism involving the reversible formation of a Cu–His^113^–Zn bridge [70]. None of the ligands investigated in the present study attached in the vicinity of the catalytic sites. SOD accommodated caffeine and theophylline in a profound cavity defined between chains A and B, whereas theobromine bound to an equivalent location in between chains C and D (Figure 11). The ligands were in direct contact with amino acids belonging to the β-strand 1 (Gln^46^), loop IV (Leu^103^) and β-strand 8 (Val^191^), found at the SOD dimer interface [70]. The formation of the complex between SOD and caffeine was favored by the hydrophobic interfaces, the most important contribution to the hydrophobic effect being assigned to Gln^48^ and Leu^103^ from chain A, Gln^48^ and Leu^103^ from chain B, and Ala^55^ from chain C of the enzyme. The positive protein affinity towards the caffeine ligand is indicated by the negative solvation free energy gain upon formation of the interface (ΔGi of −1.9 kcal/mol).

In a similar manner, SOD exerts positive affinity towards theophylline (ΔGi of −1.6 kcal/mol) and theobromine (ΔGi of −1.7 kcal/mol), with chains A and D, respectively, as main contributors (ΔGi of −1.0 kcal/mol and −0.8 kcal/mol, respectively) to the total hydrophobic interface. Both theophylline and theobromine established hydrogen bonds with Cys^189^, which has an important role in the structural organization of each enzyme subunit. In particular, the Cys^107^–Cys^189^ disulfide bond helps in connecting the loop IV with β-strand 8 and imparts the orientation of the Zn-binding loop [70].

## 4. Discussion

In the model employed in the present study, 15-day exposure of zebrafish to ZnO-NPs produced a distinct pattern of antioxidant biomarker modulation in L2, with increased GPx1, CAT, and SOD activities accompanied by depletion of GSH relative to controls.

These findings are consistent with recent research employing molecular docking approaches to investigate the interaction of ZnO-NPs with antioxidant enzymes [32]. The mechanism proposed is direct enzymatic inhibition: ZnO-NPs can bind within the catalytic sites of SOD1 and CAT via hydrogen bonds and electrostatic interactions, thereby reducing substrate accessibility and compromising catalytic efficiency [32,41]. In this context, the marked increase in SOD and CAT protein levels observed in our study may be interpreted as a compensatory response to ZnO-NPs’ oxidative pressure, consistent with the hypothesis that ZnO-NPs can impair catalytic efficiency while triggering upregulation of antioxidant defenses.

The molecular interactions between ZnO-NPs and antioxidant enzymes are not uniform and result in distinct patterns, reflected by the marked SOD induction observed in the present study, where SOD emerged as the most responsive biomarker. This differential response pattern is supported by mechanistic evidence from the recent literature as follows.

In the case of SOD, the direct interaction of nanoparticles with the catalytic site reduces reaction efficiency, resulting in compensatory induction [32]. By contrast, CAT, whose enzymatic mechanism relies on a heme group and is not directly dependent on Zn^2+^, is less affected. Its activation occurs mainly when H_2_O_2_ levels become sufficiently elevated, and regulation is achieved both through transcriptional mechanisms (Nrf2) and post-translational modifications. As a result, its response is slower and of lower magnitude [75].

The findings of Saddick et al. (2015) in *Oreochromis niloticus* and *Tilapia zillii* exposed to ZnNPs for 15 days confirm the same differential response pattern between SOD and CAT as that observed in the present study [76]. At a moderate concentration (500 µg/L), SOD activity increased by approximately 50% in both species, whereas CAT showed only modest increases (+4% to +19%). At higher, toxic concentrations (2000 µg/L), the pattern was reversed: both enzymes were inhibited, but CAT was more severely affected (−33% and −64%), whereas SOD activity decreased to a lesser extent (−39% and −49%). These results indicate that at moderate exposures, SOD is the more sensitive marker, showing stronger induction than CAT, whereas under oxidative stress conditions CAT becomes markedly more impaired. Despite methodological differences, both our data and those of Saddick et al. converge on the same response pattern, highlighting SOD as the more sensitive marker compared to CAT under ZnNPs exposure.

The diversity of experimental models reported in the literature results in a wide range of redox responses, depending on species, cell type, and exposure conditions. For instance, Dubey et al. investigated a gill cell line from Wallago attu exposed to ZnO-NPs and reported a much stronger stimulation of catalase than SOD at low doses (CAT +135% vs. SOD +26%), followed by a more severe inhibition of CAT than SOD at higher concentrations [42].

In addition to CAT and SOD, the analysis of GPx1 and GSH provides further insight into the non-enzymatic and glutathione-dependent components of the antioxidant response. GPx1 showed a more moderate shift in the group exposed to Zn-NPs (L2) compared to the other enzymatic markers, consistent with an adaptive response. GSH levels decreased by 58.7% in L2, reflecting thiol depletion under Zn-NPs exposure. The alterations in GSH observed in the present study are consistent with previous reports on metallic nanoparticles, as well as acute exposures in both embryonic stages and adult fish [48,57].

Other studies have also reported a reduction in reduced glutathione, although oxidative stress responses vary depending on the model and exposure dose. For example, Dubey et al. (2015) showed in a gill cell line of Wallago attu a compensatory increase in GSH at 12.5 mg/L ZnO-NPs (up to 155% of control), followed by marked decreases at higher doses of 25 and 50 mg/L (52% and 24% of control, respectively) [42]. This pattern highlights a biphasic response characterized by initial stimulation of antioxidant mechanisms at low concentrations, followed by severe depletion of glutathione reserves at higher concentrations.

Complementary results were recently reported in zebrafish exposed to ZnO-NPs and Ag-NPs, where after 7–14 days of exposure, statistically significant but transient increases in SOD, CAT, and GPx activities were observed, with moderate amplitudes (approximately 1.3–1.6-fold compared with controls). In parallel, GSH levels were reduced in a clear dose-dependent manner. The most marked effects were recorded under co-exposure to ZnO-NPs and Ag-NPs, where both enzymatic induction and GSH depletion were enhanced, indicating a potential synergistic disruption of redox homeostasis [43]. Although the cited data refer to enzymatic activities, whereas the present study assessed biomarker levels, the convergence of the response pattern—antioxidant enzyme upregulation combined with GSH depletion—supports the consistency of this mechanism in fish exposed to metallic nanoparticles.

The literature indicates that not only ZnO-NPs but also other metallic nanoparticles, such as TiO_2_, Ar, or SiO, induce similar alterations in redox balance, characterized by an initial stimulation of antioxidant enzymes followed by inhibition of their activity and expression under prolonged exposure [43,77,78].

Several studies emphasize that the modulation of antioxidant enzymes is largely dependent on the duration of exposure. For instance, in zebrafish chronically exposed to ZnO-NPs and Ag-NPs, transient increases in SOD and CAT activity were observed at 7–14 days, followed by significant inhibition at 28 days, suggesting exhaustion of enzymatic defenses after prolonged exposure. GPx induction was modest and mainly observed under co-exposure conditions, while long-term exposure led to decreased activity accompanied by progressive depletion of glutathione reserves [43]. Similarly, Tang et al. reported that zebrafish exposed to TiO_2_ nanoparticles for 28 days exhibited significantly reduced SOD and CAT activity in the liver and gills, reflecting exhaustion of antioxidant defenses and increased susceptibility to oxidative injury [78].

Co-exposure to methylxanthines and ZnO-NPs markedly reduced the GPX1 elevation observed under nanoparticle exposure alone (L2), with theobromine producing the most pronounced effect, followed by caffeine and theophylline. This pattern was consistently supported by non-parametric multigroup comparison tests (*p* < 0.001–0.01), clustering, and regression analyses, indicating that GPX1 is particularly responsive to methylxanthine modulation in the presence of ZnO-NPs.

Glutathione levels showed a similar pattern. While ZnO-NP exposure in group 2 was associated with a depletion of GSH, co-administration of methylxanthines partially restored thiol reserves. This trend was supported by group comparison tests and regression analyses, which identified theobromine as the most potent enhancer of GSH in the presence of ZnO-NP (β = +1.559, *p* = 0.002), followed by caffeine, while theophylline exerted only a minimal influence.

Regarding the antioxidant enzyme components SOD and CAT, differences were also observed between groups, reflecting compound-specific responses. While exposure to ZnO-NP (L2) alone was associated with increased CAT levels, co-exposure to methylxanthines diminished this response to varying degrees, with caffeine exerting the strongest influence, followed by theobromine and theophylline. These relationships were demonstrated by ANOVA comparison analysis (*p* < 0.05) and regression models, indicating that CAT is particularly sensitive to caffeine under conditions of nanoparticle exposure (β = −1.10, *p* < 0.05).

SOD showed a comparable pattern. Although ZnO-NP exposure increased SOD activity, co-administration of methylxanthines reduced this response, with theophylline exerting the strongest influence, followed by caffeine and theobromine.

While conventional between-group comparison tests identified a significant reduction in SOD only in the caffeine co-exposure group (*p* = 0.009), regression modeling revealed a specific interaction pattern, showing that all three methylxanthines exerted inhibitory effects on SOD in the presence of ZnO nanoparticles.

This response pattern suggests that methylxanthine administration mitigates the ZnO-NPs exposure effect of antioxidant enzymes’ hyper-induction and contributes to the restoration of redox homeostasis, with compound-specific protective effects: caffeine exerted the strongest influence on CAT/SOD, theobromine on GSH/GPx1, and theophylline displayed only minor effects.

In groups exposed exclusively to methylxanthines (L6–L8), SOD and CAT activities increased relative to the control group, while GPx1 levels were lower than in the control group and GSH remained essentially unchanged. This profile indicates that methylxanthines alone can activate enzymatic antioxidant pathways, without depleting thiol pools. Among the compounds tested, caffeine elicited more consistent effects on SOD and CAT, while theobromine and theophylline produced progressively milder responses, reflecting distinct regulatory influences on antioxidant components.

PCA further supported the distinct biochemical profiles identified across exposure groups. GPx1 showed an inverse association with GSH, while CAT and SOD were positively correlated, indicating a functional balance between enzymatic and non-enzymatic antioxidant defenses. ZnO-NP exposure clustered in the quadrant defined by increased GPx1, CAT, and SOD and GSH depletion, consistent with an oxidative challenge. Methylxanthines shifted scores toward +F1 and lower F2 values, indicating partial restoration of GSH and lower enzyme levels in L3–L5 compared with the ZnO-NP exposure group L2; methylxanthine-only groups (L6–L8) were located closer to the control group, with moderate CAT/SOD activation and no GSH depletion.

Although methylxanthines have been extensively studied in neurological and metabolic contexts in mammals, data on their effects on redox balance in aquatic vertebrate models exposed to oxidative stress with zinc oxide nanoparticles (ZnO-NPs) remain limited. Several studies have reported that xanthines can mitigate oxidative stress by moderately enhancing SOD activity and reducing malondialdehyde (MDA) levels in animals, yet the underlying mechanisms are not fully elucidated [79,80].

Electrochemical and spectroscopic investigations by Petrucci et al. (2018) [81] demonstrated that methylxanthines interact with reactive oxygen species through an electron-transfer mechanism rather than classical hydrogen-atom donation. Oxidation occurs predominantly at the C4 = C5 double bond, producing electronically stabilized cationic radicals for N7-methylated compounds such as caffeine and theobromine, and neutral radicals for theophylline. These stabilized species are relatively unreactive, thereby preventing the formation of secondary radicals and interrupting oxidative chain reactions. As a result, methylxanthines exert antioxidant effects primarily through redox modulation rather than direct radical scavenging [81].

Recent work by Vieira et al. (2020) [50] provides complementary evidence for this mechanism. Using adenine as a biological target and hydroxyl radicals as oxidants, the authors showed that caffeine does not behave as a classical scavenger, since its reaction rate matches that of adenine and it cannot regenerate adenine from its radical form. Instead, caffeine is oxidized to secondary products such as trimethyluric acid, theobromine, and theophylline, which possess genuine antioxidant properties. This cascade effect explains the delayed yet significant protection against oxidative damage, indicating that caffeine acts indirectly through its transformation products rather than through intrinsic radical-scavenging capacity [50].

Beyond these allosteric interactions, methylxanthines exert indirect antioxidant effects by modulating signaling pathways. They act as antagonists of adenosine receptors (A_1_, A_2_A), induce Nrf2 and HO-1 expression, and inhibit phosphodiesterases (PDEs), thereby increasing cAMP levels and PKA activity, which negatively regulate NF-κB [74]. Through these mechanisms, methylxanthines can upregulate antioxidant enzyme expression, maintain redox homeostasis, and reduce lipid peroxidation. However, Sitarek et al. (2024) showed that theobromine exerts antioxidant effects in normal cells under oxidative stress but can act pro-oxidatively and pro-apoptotically in tumor cells via NF-κB, PDE4, and MAPK modulation, highlighting a dual, context-dependent role [82].

Consistent with these mechanisms, the moderate increase in SOD and CAT observed in our study in groups exposed exclusively to methylxanthines (L6–L8) likely reflects a mild stimulatory effect on endogenous antioxidant defenses. Although methylxanthines mitigated several biochemical changes induced by ZnO-NP exposure, their effects did not follow a uniform antioxidant pattern, which could indicate different modulation of redox pathways rather than a common protective mechanism. Theobromine, for example, significantly reduced GPx1 levels while restoring GSH, indicating that its action may rely more on thiol replenishment than GPx1-dependent peroxide detoxification. In contrast, caffeine exerted stronger effects on CAT and SOD, consistent with its reported influence on mitochondrial and Nrf2-related signaling, while theophylline produced only modest changes between markers. These divergent profiles highlight that methylxanthines do not function as direct-acting antioxidants, but rather as compounds with compound-specific redox modulation.

The results of the molecular docking tests revealed that all tested methylxanthines are able to recognize and attach to different cavities of the antioxidant enzymes. The interaction surface of all complexes involving theophylline and theobromine as ligands appeared to be stabilized through hydrogen bonds and π-stacking interactions, which might significantly contribute to the stabilization of enzymes.

The detailed analysis of molecular models of the docking complexes involving GPx1 as the receptor indicated no direct involvement of the amino acids from the active site in the interaction with caffeine, theobromine, or theophylline. In addition, the allosteric modulation of the enzyme activity upon the ligand binding to a different substrate should not be neglected. Kosksi et al. (2025) indicated that binding of the allosteric modulators might result in important improvement of the efficiency and/or enzyme stability, as a consequence of conformational rearrangements [83].

In the case of CAT, the molecular docking results suggested that ligands binding might interfere with enzymes activity because of the direct involvement in the interactions of three amino acids positioned in the close vicinity of the active site heme. In particular, all ligands directly interface Arg^363^ and His^364^, which are in the neighborhoods of Arg^365^ residues, with an important role in the stabilization of the active site conformation. The salt bridges between the heme carboxylate radical and Arg^72^, Arg^117^, and Arg^365^ are crucial for heme burial within protein chains, contributing to the local redox potential increase [74]. Caffeine and theobromine additionally interact with Asp^360^, which is one residue apart from Tyr^358^, at a distance of ~1.85Å from heme N-Fe [74]. Therefore, binding of the studied ligands to the CAT molecule might critically interfere with the functionality of the heme active site, as Tyr^358^ is involved in iron heme tuned by electron donation, while Arg^365^ contributes to the neutralization of the carboxylate charge, together with Arg^72^ and Arg^112^. Even small local rearrangement caused by ligands binding to CAT could affect substrate recognition and/or transformation.

Regarding the molecular docking models of the complexes involving SOD as the receptor, our results comply with the observation of Wu et al. (2019) [80], who showed that theophylline and theobromine are accommodated at the interface of the enzyme subunits [80]. None of the molecular docking complexes described by Wu et al. (2019) [80] involved the catalytic site of the enzyme in binding the ligand. Based on the binding energy values, enzyme–ligand complex formation was more favorable in the case of theophylline (−27.48 kJ/mol) compared to theobromine (−23.73 kJ/mol). The authors indicated that the complexes were stabilized through hydrogen bonds, alkyl, and π-alkyl interactions. The slightly higher affinity noted in the case of the complex with theophylline was assigned to a π-donor interaction. Our results are in agreement with Wu et al. (2019) [80], suggesting that ligands interact with SOD in an allosteric manner. Wu et al. (2019) [80] additionally suggested that the enzymatic activity remains unchanged upon theophylline and theobromine, supporting the experimental observations which indicated that their antioxidant effects do not arise from a direct modification of the enzyme’s catalysis [80].

Liu et al. (2019) [84] investigated the interaction between caffeine and Cu/Zn-SOD using a combination of spectroscopic and molecular modeling techniques [84]. The authors demonstrated that caffeine specifically interacts with Cu/Zn-SOD, resulting in complexes stabilized by hydrogen bonds and van der Waals forces, with spontaneous binding energies of −26 kJ/mol. The interaction involved an enzyme cavity distinct from the active metal site, explaining why the enzyme’s catalytic activity was not affected, although UV and fluorescence spectroscopy indicated slight conformational changes. The SOD–caffeine complex formation appeared to stabilize the enzyme, enhancing its overall antioxidant capacity through a synergistic effect on free radicals [84].

### Limitations of the Study

Although this study assessed a limited set of enzymatic (CAT, SOD, GPx1) and non-enzymatic (GSH) markers, these measurements were intended to assess early systemic redox responses. Future investigations integrating the quantification of reactive oxygen species (ROS) and molecular criteria for assessing oxidative damage may provide a more comprehensive perspective on ZnO-NP-induced oxidative stress.

One limitation of the molecular docking investigation consists on the use of human enzymes, due to the lack of molecular models of enzymes originating from zebrafish. Therefore, one should also factor in the potential differences in terms of key amino acids when considering the mechanistic evidence regarding the effects of methylxanthines binding on the antioxidant enzymes’ functionality.

## 5. Conclusions

The concomitant assessment of GSH, SOD, CAT, and GPx1 in zebrafish provided an integrated perspective on how these molecules respond to a pro-oxidant challenge of zinc oxide nanoparticle (ZnO-NP) exposure and how methylxanthines modulate this biochemical profile. To address this, robust statistical analyses (post hoc comparisons, interaction regression models, correlations, and PCA) were applied, enabling the distinct quantification of the effects of ZnO-NP exposure and of each methylxanthine’s contribution within the zebrafish model.

The results demonstrated that 15-day exposure to ZnO-NPs induced marked alterations of antioxidant biomarkers, characterized by hyper-induction of antioxidant enzymes (most notably SOD, followed by CAT and GPx1) and significant depletion of reduced glutathione.

The findings indicate that methylxanthines exerted a protective effect by reducing ROS levels and modulating redox-related pathways, thereby lowering the need for an intense compensatory antioxidant response. Compound-specific differences were also observed: theobromine showed the strongest effect on restoring glutathione balance (reduced GPx1 and replenished GSH) and caffeine was the most effective modulator of catalase (attenuating excessive induction), while theophylline displayed a weaker protective profile, with comparatively modest effects on both enzymatic and non-enzymatic markers.

Finally, the molecular docking highlighted the binding affinity of the tested methylxanthines towards the antioxidant enzymes, influencing in different manners their functionality. Theophylline and theobromine appeared to exert a modulatory role upon complexing the GPx1 and SOD molecules. Because of the strong attachment in the close vicinity of heme binding sites, the ligands binding might particularly interfere with CAT activity.

## Figures and Tables

**Table 1:** Experimental group’s overview.

Group	ZnO-NPs	Xanthine Compound
1 (Control)	–	–
2	0.69 mg/L	–
3	0.69 mg/L	Caffeine 50 mg/L
4	0.69 mg/L	Theophylline 50 mg/L
5	0.69 mg/L	Theobromine 50 mg/L
6	–	Caffeine 50 mg/L
7	–	Theophylline 50 mg/L
8	–	Theobromine 50 mg/L

**Table 2:** Results of the analyzed parameters by experimental groups; data are expressed as mean ± standard deviation (SD) for SOD (normally distributed) and as median with interquartile range (IQR) for GPX1, glutathione, and catalase (non-normally distributed).

Group	GPX1 pg/g	CAT pg/g	SOD ng/g	GSH µg/g
L1 control	84.270 (79.45–86.19)	38.255 ± 10.43	0.122 (0.10–0.13)	2.900 (2.80–2.98)
L2 (ZnO-NPs)	142.240 (112.64–163.67)	100.756 ± 19.43	1.090 (0.93–1.24)	1.200 (0.96–1.43)
L3 (ZnO-NPs + caffeine)	65.240 (59.28–73.54)	71.173 ± 19.06	0.490 (0.38–0.61)	1.670 (1.11–1.80)
L4 (ZnO-NPs + theophylline)	79.420 (69.01–88.57)	82.082 ± 21.46	0.650 (0.47–0.76)	2.000 (1.70–2.43)
L5 (ZnO-NPs + theobromine)	25.030 (23.14–37.96)	76.012 ± 20.37	0.568 (0.31–0.95)	3.040 (2.47–4.70)
L6 (caffeine)	67.435 (54.20–80.72)	90.760 ± 19.27	0.820 (0.41–1.07)	2.118 (1.99–2.98)
L7 (theophylline)	51.070 (41.40–59.52)	78.551 ± 20.52	0.570 (0.17–0.61)	3.070 (2.39–3.95)
L8 (theobromine)	52.340 (47.89–65.05)	76.455 ± 14.55	0.470 (0.27–0.54)	3.170 (2.57–4.08)

**Table 3:** Details on the interaction between the antioxidant enzymes, namely glutathione peroxidase 1 (GPx1), catalase (CAT), and superoxide dismutase (SOD), and ligand molecules consisting of caffeine, theophylline, and theobromine.

	**Complexes formed between GPx1 and**		
	**caffeine**	**theophylline**	**theobromine**
Binding energy, kcal/mol	−4.219	−4.292	−4.262
Interaction surface, Å^2^	78.6	132.2	124.4
Interfacing residues	Chain B: Ala^77^, Gln^78^, His^81^, Asn^84^, Lys^112^, Glu^114^	Chain A: Asp^144^, Lys^146^, Leu^147^ Chain B: Asn^77^, Gln^78^, His^81^, Glu^114^, Thr^149^	Chain A: His^81^, Thr^149^ Chain B: Asp^144^, Lys^146^, Leu^147^
Amino acids (chain) involved in interactions with the ligands	-	H bonds: Thr^149^ (B)	H bonds: Thr^149^ (A) π-stacking interactions: His^81^ (A)
	**Complexes formed between CAT and**		
	**caffeine**	**theophylline**	**theobromine**
Binding energy, kcal/mol	−5.350	−5.522	−5.609
Interaction surface, Å^2^	171.5	174.1	173.3
Interfacing residues	Chain A: Asp^360^, Arg^363^, His^364^, Pro^368^ Chain B: Arg^66^ Chain C: Asp^360^, Arg^363^, His^364^, Pro^368^, Pro^391^ Chain D: Arg^66^	Chain B: Arg^66^ Chain C: Arg^363^, His^364^, Pro^368^ Chain D: Arg^66^	Chain A: Asp^360^, Arg^363^, His^364^, Pro^368^ Chain B: Arg^66^ Chain C: Asp^360^, Arg^363^, His^364^, Pro^368^ Chain D: Arg^66^
Amino acids (chain) involved in interactions with the ligands	-	H bonds: Arg^66^ (B)	H bonds: His^364^ (C), Arg^66^ (D)
	**Complexes formed between SOD and**		
	**caffeine**	**theophylline**	**theobromine**
Binding energy, kcal/mol	−5.614	−5.376	−5.387
Interaction surface, Å^2^	143.4	165.1	139.0
Interfacing residues	Chain A: Gln^46^, Gln^48^, Leu^103^ Chain B: Gln^46^, Gln^48^, Pro^49^, Leu^103^, Cys^189^, Cys^190^ Chain C: Ala^55^	Chain A: Gln^46^, Gln^48^, Leu^103^, Cys^189^, Cys^190^, Val^191^ Chain B: Cys^45^, Gln^46^, Gln^48^, Leu^103^, Cys^190^, Val^191^	Chain B: Ala^55^ Chain C: Gln^46^, Gln^48^, Leu^103^, Cys^190^ Chain D: Gln^46^, Gln^48^, Leu^103^, Cys^189^, Cys^190^, Val^191^
Amino acids (chain) involved in interactions with the ligands	-	2 H bonds: Cys^189^ (A)	H bonds: Cys^189^ (D)

## Data Availability

All data supporting the findings of this study are included within the article and its Supplementary Materials.

## Supplementary Materials

The following supporting information can be downloaded at https://www.mdpi.com/article/10.3390/medicina62010021/s1, Figure S1: Heatmap Spearman correlation matrix of antioxidant markers (GPX1, glutathione, catalase, and SOD); Figure S2: Multiple linear regression analysis for GPX1 (pg/mL). Representation of standardized predictor coefficients (left) and correlation between observed and predicted values (right); Figure S3: Hierarchical clustering (Ward’s method, Euclidean distance) of analyzed biomarkers; Table S1: Post hoc analysis results (Tukey HSD and Dunnett) of catalase levels (pg/mL) across the eight experimental groups; Table S2: Spearman correlation matrix (r^ss^;) among the biochemical parameters studied (GPX1, glutathione, catalase, and SOD), with associated *p*-values. Significant correlations are highlighted in bold.
