# Supplementary material for: Evaluation of Oxidative Stress and Antioxidant Effects of Methylxanthines in Adult Zebrafish Exposed to Zinc Oxide Nanoparticles (ZnO-NPs)
Source: Medicina (Kaunas). 2025 Dec 22;62(1):21. doi: 10.3390/medicina62010021 (PMC12843068; doi:10.3390/medicina62010021)
Supplement: Supplementary file 1 [file medicina-62-00021-s001.zip › medicina-3981384-supplementary.pdf]

Supplementary material of the manuscript "Evaluation of Oxidative Stress Biomarkers and Antioxidant Effects of Natural Methylxanthines in Adult Zebrafish Exposed to Zinc Oxide Nanoparticles (ZnO-NPs)"

Materials and methods

Determination of GPx1, CAT, SOD and GSH by ELISA

1. GPx1 (Glutathione Peroxidase 1) – Sandwich ELISA

The determination of GPx1 was performed using a quantitative sandwich ELISA. Microplates were pre-coated with a monoclonal antibody specific for zebrafish GPx1. Samples were added to the wells and incubated to allow antigen binding, followed by the addition of a biotinylated detection antibody. After washing, a streptavidin–HRP conjugate was applied, generating a chromogenic signal upon reaction with TMB substrate. The enzymatic reaction was stopped with sulfuric acid, and absorbance was measured at 450 nm. GPx1 concentrations were determined from a standard curve supplied with the kit.

Detection limit: 7.2 pg/mL, enabling the quantification of low GPx1 levels with high analytical sensitivity. No cross-reactivity with related peroxidases was reported by the manufacturer.

2. CAT (Catalase) – Sandwich ELISA

Catalase concentrations were measured via a sandwich-type ELISA using microplates coated with CAT-specific antibodies. After incubation with the samples, a biotinylated secondary antibody was added to form the antigen–antibody complex. Detection was achieved through streptavidin-HRP and TMB chromogenic substrate, and absorbance was recorded at 450 nm. The CAT content of the samples was calculated relative to the standard curve provided by the manufacturer.

Detection limit: 6.5 pg/mL, ensuring reliable quantification in zebrafish homogenates.

3. SOD (Superoxide Dismutase) – Sandwich ELISA

SOD levels were quantified using ELISA plates coated with antibodies specific to zebrafish SOD. Bound antigen was detected using a biotinylated antibody and streptavidin-HRP, followed by TMB substrate and spectrophotometric reading at 450 nm. Concentrations were extrapolated from the calibration curve.

Detection limit: 0.069 ng/mL (~69 pg/mL), allowing detection of SOD in low-abundance biological samples.

4. GSH (Reduced Glutathione) – Competitive ELISA

Reduced glutathione was determined using a competitive ELISA, in which GSH in the sample competes with plate-bound GSH for a limited amount of biotinylated antibody. After binding and washing steps, detection was performed using a streptavidin-HRP complex and TMB substrate. The color intensity is inversely proportional to the GSH concentration in the sample. Absorbance was read at 450 nm, and concentrations were interpolated from the standard curve supplied in the kit.

Detection limit: 0.41 μg/mL, allowing sensitive quantification of glutathione variation in zebrafish tissues.

Table 1S. Post hoc analysis results (Tukey HSD and Dunnett) of catalase levels (pg/mL) across the eight experimental groups

Contrast	Differences	p value	Contrast	Differences	p value
L2 vs L1	62.500	**<0.0001**	L6 vs L1	52.505	**<0.0001**
L2 vs L3	29.583	**0.001**	L6 vs L3	19.587	0.081
L2 vs L5	24.743	**0.009**	L6 vs L5	14.748	0.368
L2 vs L8	24.300	**0.011**	L6 vs L8	14.305	0.408
L2 vs L7	22.205	**0.028**	L6 vs L7	12.209	0.614
L2 vs L4	18.674	0.114	L6 vs L4	8.678	0.902
L2 vs L6	9.996	0.815	L7 vs L1	40.296	**<0.0001**
L3 vs L1	32.918	**<0.0001**	L7 vs L3	7.378	0.957
L4 vs L1	43.827	**<0.0001**	L7 vs L5	2.539	1.000
L4 vs L3	10.909	0.739	L7 vs L8	2.096	1.000
L4 vs L5	6.069	0.986	L8 vs L1	38.200	**<0.0001**
L4 vs L8	5.627	0.991	L8 vs L3	5.282	0.994
L4 vs L7	3.531	1.000	L8 vs L5	0.443	1.000
L5 vs L1	37.757	**<0.0001**			
L5 vs L3	4.840	0.996			

Table 2S. Spearman correlation matrix (r_s) among the biochemical parameters studied (GPX1, glutathione, catalase, and SOD), with associated p-values. Significant correlations are highlighted in bold

**Variable**	**GPX1**	**GSH**	**CAT**	**SOD**
GPX1	1	**r = –0.482, p < 0.0001**	r = 0.060, p = 0.517	r = 0.151, p = 0.100
GSH	**r = –0.482, p <0.0001**	**1**	**r = –0.190, p = 0.038**	**r = –0.374, p < 0.0001**
CAT	r = 0.060, p 0.517	**r = –0.190, p = 0.038**	1	**r = 0.399, p < 0.0001**
SOD	r = 0.151, p = 0.100	**r = –0.374, p < 0.0001**	**r = 0.399, p < 0.0001**	1

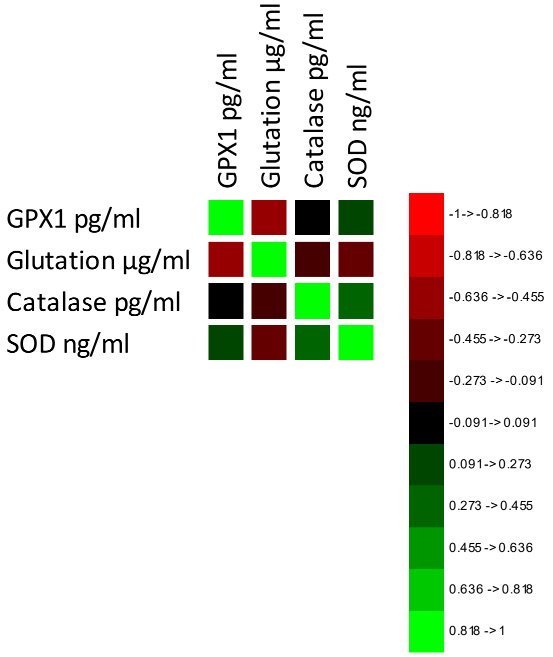

Figure 1S. Heatmap Spearman correlation matrix of antioxidant markers (GPX1, glutathione, catalase, and SOD)

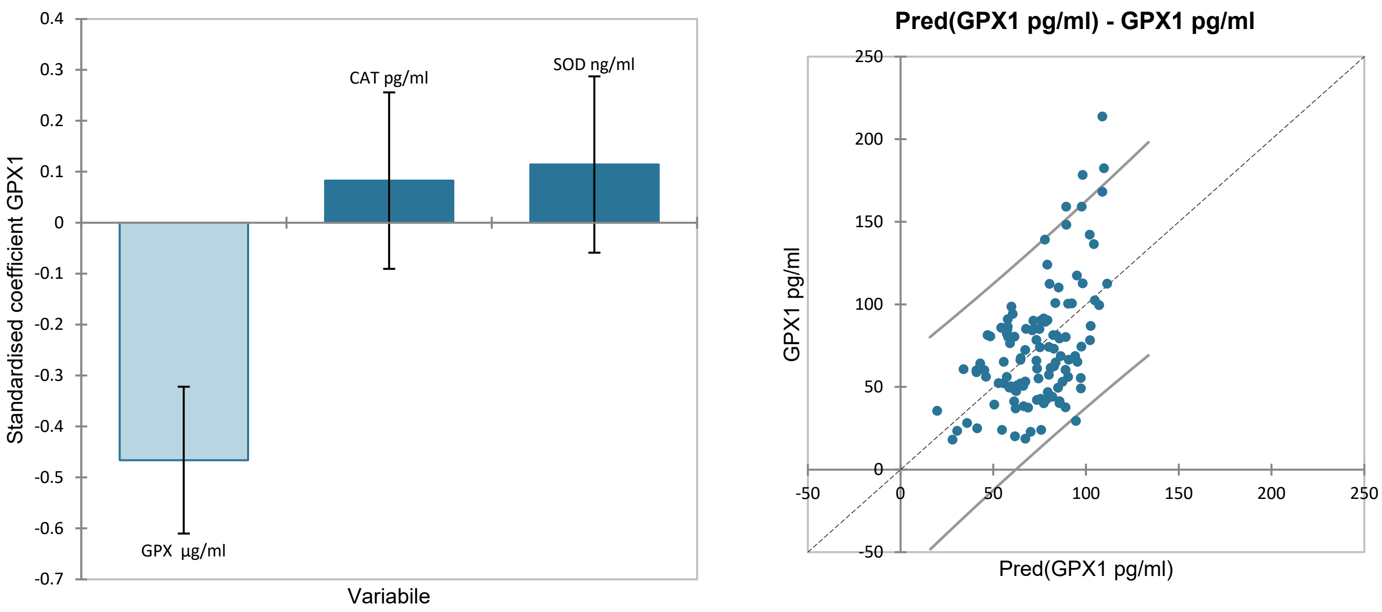

Figure 2S. Multiple linear regression analysis for GPX1 (pg/mL). Representation of standardized predictor coefficients (left) and correlation between observed and predicted values (right).

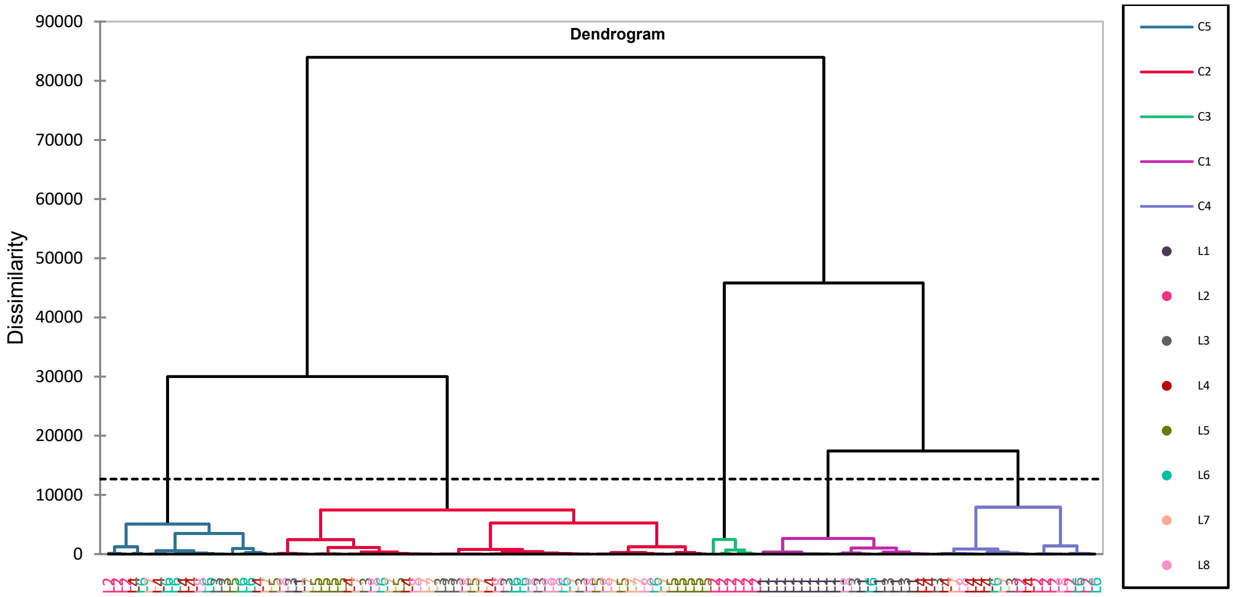

Figure 3S. Hierarchical clustering (Ward's method, Euclidean distance) of analyzed biomarkers
